# A LC3-Interacting Motif in the Influenza A Virus M2 Protein Is Required to Subvert Autophagy and Maintain Virion Stability

**DOI:** 10.1016/j.chom.2014.01.006

**Published:** 2014-02-12

**Authors:** Rupert Beale, Helen Wise, Amanda Stuart, Benjamin J. Ravenhill, Paul Digard, Felix Randow

**Affiliations:** 1 MRC Laboratory of Molecular Biology, Francis Crick Avenue, Cambridge CB2 0QH, UK; 2 University of Cambridge, Department of Medicine, Addenbrooke’s Hospital, Cambridge CB2 0QQ, UK; 3 Roslin Institute, University of Edinburgh, Edinburgh EH25 9RG, UK; 4 Division of Virology, Department of Pathology, University of Cambridge, Cambridge CB2 0QQ, UK

## Abstract

Autophagy recycles cellular components and defends cells against intracellular pathogens. While viruses must evade autophagocytic destruction, some viruses can also subvert autophagy for their own benefit. The ability of influenza A virus (IAV) to evade autophagy depends on the Matrix 2 (M2) ion-channel protein. We show that the cytoplasmic tail of IAV M2 interacts directly with the essential autophagy protein LC3 and promotes LC3 relocalization to the unexpected destination of the plasma membrane. LC3 binding is mediated by a highly conserved LC3-interacting region (LIR) in M2. The M2 LIR is required for LC3 redistribution to the plasma membrane in virus-infected cells. Mutations in M2 that abolish LC3 binding interfere with filamentous budding and reduce virion stability. IAV therefore subverts autophagy by mimicking a host short linear protein-protein interaction motif. This strategy may facilitate transmission of infection between organisms by enhancing the stability of viral progeny.

## Highlights

• Influenza A misappropriates autophagy via its M2 proton channel
• M2 binds the essential autophagy protein LC3 via a highly conserved LIR motif
• The M2 LIR motif relocalizes LC3 to the plasma membrane
• Mutations in the LIR motif affect filamentous budding and reduce stability of virus

## Graphical Abstract

**Figure:**
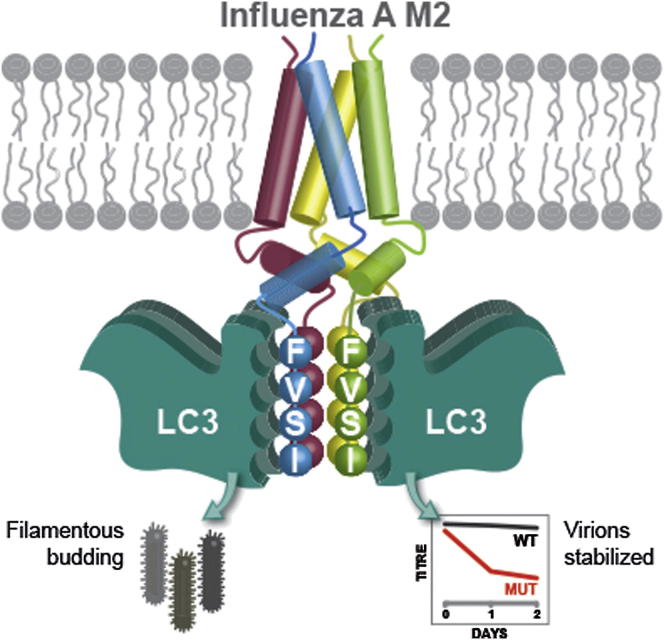

## Teaser

Beale et al. discover that the influenza A virus M2 protein contains a cytoplasmic LC3-interacting region (LIR) motif that binds to the autophagy protein LC3 and diverts it to the plasma membrane during virus budding. The LIR motif subverts autophagy and is required to promote filamentous budding and virion stability.

## Introduction

Macroautophagy (hereafter autophagy) can generate raw materials at times of cellular stress by degrading cytoplasmic contents (Mizushima and Komatsu, 2011). During starvation-induced autophagy, cytoplasm is nonselectively engulfed into double-membrane vesicles (autophagosomes), whose content is digested upon fusion with lysosomes. This process has been adapted for host defense, where invading pathogens are selectively targeted by autophagy (Deretic, 2012; Levine et al., 2011; Randow et al., 2013). Selective antimicrobial autophagy relies on cargo receptors that simultaneously detect pathogen-associated “eat me” signals and bind to members of the ATG8/LC3 family of ubiquitin-related proteins residing on autophagosomal membranes (Boyle and Randow, 2013; Thurston et al., 2009; 2012; Wild et al., 2011; Zheng et al., 2009). Binding partners of LC3/ATG8 family members typically contain an LC3-interacting region (LIR). LIRs form intermolecular β sheets with LC3/ATG8 family members by virtue of a consensus W/FxxI/L motif, often preceded by acidic residues (Johansen and Lamark, 2011).

While autophagy restricts the growth of pathogens poorly adapted to life in the cytosol (e.g., *Salmonella enterica*), professional cytosol-dwelling bacteria, such as *Shigella flexneri*, evade restriction by autophagy (Boyle and Randow, 2013; Mostowy and Cossart, 2012). Other pathogenic microbes have evolved mechanisms to actively benefit from host autophagy. Multiple viruses deploy such subversion strategies, but the molecular mechanisms involved are unclear (Feng et al., 2013; Heaton and Randall, 2010; Klein and Jackson, 2011; Reggiori et al., 2010).

The sizes of RNA virus genomes are evolutionarily constrained (Belshaw et al., 2008). Their need to hijack cellular machinery while evading host immune responses requires complex coding capacities and multifunctional viral proteins. Multiple distinct peptide products are often produced from the same gene (e.g., Firth and Brierley, 2012; Thomas et al., 1988; Wise et al., 2012). In addition, viral proteins subvert host physiology by encoding short linear motifs (SLiMs) that mimic host protein interaction interfaces, but which require little coding capacity (Davey et al., 2011). No viral SLiMs have yet been described to subvert autophagy.

One important human pathogen affecting host cell autophagy is influenza A virus (IAV). This enveloped, segmented negative sense RNA virus infects a wide range of vertebrate species and causes seasonal epidemics and sporadic pandemics in humans as well as outbreaks in domestic animals. Epidemic IAV causes significant annual global mortality and in pandemic years can result in millions of deaths (Taubenberger and Kash, 2010).

The ability of IAV to subvert autophagy is dependent on the Matrix 2 (M2) ion-channel protein, which blocks fusion of autophagosomes with lysosomes (Gannagé et al., 2009). M2 is a tetrameric integral membrane protein, made from a spliced transcript (Holsinger et al., 1994; Lamb et al., 1985). M2 comprises a 25 amino acid N-terminal ectodomain, a transmembrane α helix and a 50 amino acid cytoplasmic domain. This cytoplasmic tail contains a membrane-proximal amphipathic α helix and a membrane-distal region of unknown structure (Schnell and Chou, 2008; Sharma et al., 2010; Stouffer et al., 2008). M2 has multiple important roles in the virus life cycle. During viral entry its ion channel activity is required to trigger disassembly in response to lowered endosomal pH, and its cytoplasmic tail contributes to virus assembly, budding, and morphogenesis (Rossman and Lamb, 2011).

Here, we report a LIR motif in the cytoplasmic tail of M2. The M2 LIR motif causes the relocalization of LC3 to the plasma membrane in IAV-infected cells at the time of virus budding, and is essential to generate stable viral progeny. We propose that in addition to blocking autophagosome maturation (Gannagé et al., 2009), IAV hijacks the autophagy machinery via the M2 LIR motif to provide suitable resources for viral budding and to enhance virion stability.

## Results

### Infection with Influenza A Virus Redirects LC3 to the Plasma Membrane

**Figure 1:**
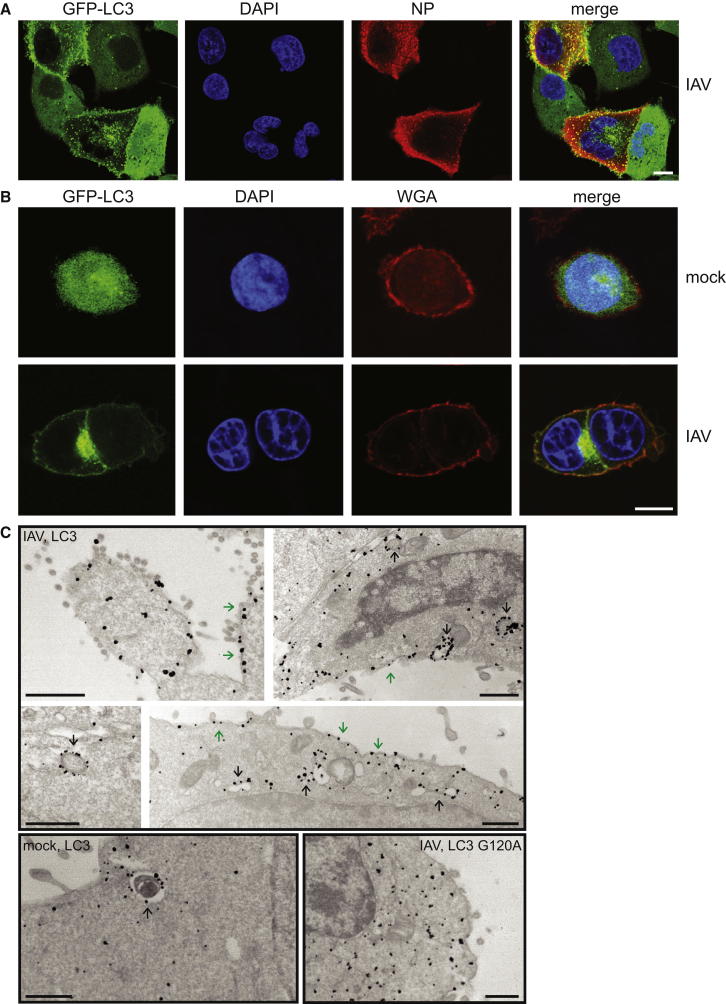
Relocalization of LC3 to the Plasma Membrane in Influenza-Infected Cells (A) A549 cells expressing GFP-LC3 were infected with PR8 strain IAV at an moi of 0.2, fixed at 16 hr p.i., and stained with anti-NP. Scale bar, 10 μm. (B) HCT116 cells expressing GFP-LC3 were infected as above and stained with WGA. Scale bar, 10 μm. (C) HCT116 cells expressing the indicated LC3 alleles fused to GFP were infected with IAV, fixed at 16 hr p.i., and stained with gold-labeled anti-GFP antibody. Black arrows, autophagosomes or autophagolysosomes; green arrows, labeling of plasma membrane. Scale bars, 500 nm. See also Figure S1.

To observe how IAV subverts the cellular autophagocytic machinery, we investigated the distribution of GFP-tagged LC3 upon virus infection. Human cell lines, stably transduced with GFP-LC3, were infected with IAV strain A/PR/8/34 (H1N1) (Figure 1A and Figure S1 available online). In uninfected cells, GFP-LC3 was distributed diffusely throughout the cytoplasm. Following infection, LC3 accumulated in the perinuclear region, as previously described (Gannagé et al., 2009). In addition, GFP-LC3 strikingly relocalized to the cell periphery. To investigate the subcellular localization of LC3 in infected cells, we stained nonpermeabilized cells with wheat-germ agglutinin (WGA) to label extracellular glycans. In uninfected cells, there was little colocalization of GFP-LC3 and WGA, while in infected cells GFP-LC3 and WGA colocalized at the plasma membrane, but not on perinuclear autophagosomes (Figure 1B). To verify the presence of LC3 at the plasma membrane and examine the subcellular distribution of autophagosomes in IAV-infected cells, we performed immunoelectron microscopy (Kabeya et al., 2000). As expected, in both mock and IAV-infected cells, LC3 was present in double-membrane vesicles corresponding to autophagosomes (Figure 1C). Infected but not uninfected cells also demonstrated staining of the plasma membrane, both at sites of active viral budding and in areas where less budding was taking place (Figure 1C). LC3 G120A, a mutant that cannot be lipidated, did not localize to autophagosomes or the plasma membrane. Taken together, these data demonstrate that IAV directs LC3 to the plasma membrane, an unusual and unexpected destination.

### Influenza A Virus M2 Protein Drives Plasma Membrane Localization of LC3

**Figure 2:**
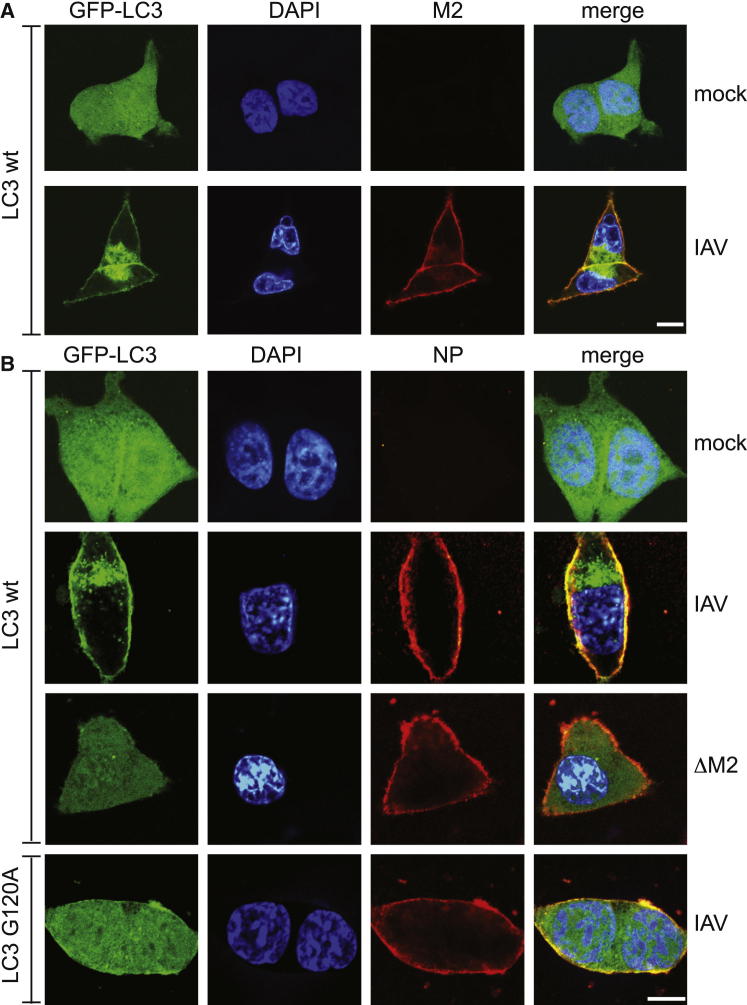
Relocalization of LC3 to the Plasma Membrane Requires Influenza M2 (A) HCT116 cells expressing GFP-LC3 were infected with IAV, fixed at 16 hr p.i., and stained with anti-M2. Scale bar, 10 μm. (B) HCT116 cells expressing the indicated LC3 alleles fused to GFP were infected with WT or ΔM2 mutant IAV, fixed at 16 hr p.i., and stained with anti-NP. Scale bar, 10 μm. See also Figure S1.

The pattern of LC3 immunogold staining (Figure 1C) parallels the known distribution of M2, which, though abundant at the plasma membrane during budding, is incorporated into virions at low levels (Leser and Lamb, 2005). Furthermore, GFP-LC3 and M2 closely colocalized at the plasma membrane (Figures 2A and S1). This finding prompted us to test whether M2 is required for the relocalization of LC3. In contrast to wild-type (WT) IAV, infection with a virus deficient in M2 (V7-T9; named here ΔM2) due to mutation of the M2 splice donor site (Hutchinson et al., 2008; Wise et al., 2012) did not relocalize LC3 to the plasma membrane or induce its perinuclear accumulation (Figure 2B). GFP-LC3 G120A failed to relocalize in cells infected with WT virus (Figure 2B), confirming the electron microscopy data (Figure 1C) and indicating that the autophagy machinery is required for IAV to redistribute LC3 in infected cells.

### Influenza M2 Contains a Conserved LIR Motif in Its Cytoplasmic Tail

**Figure 3:**
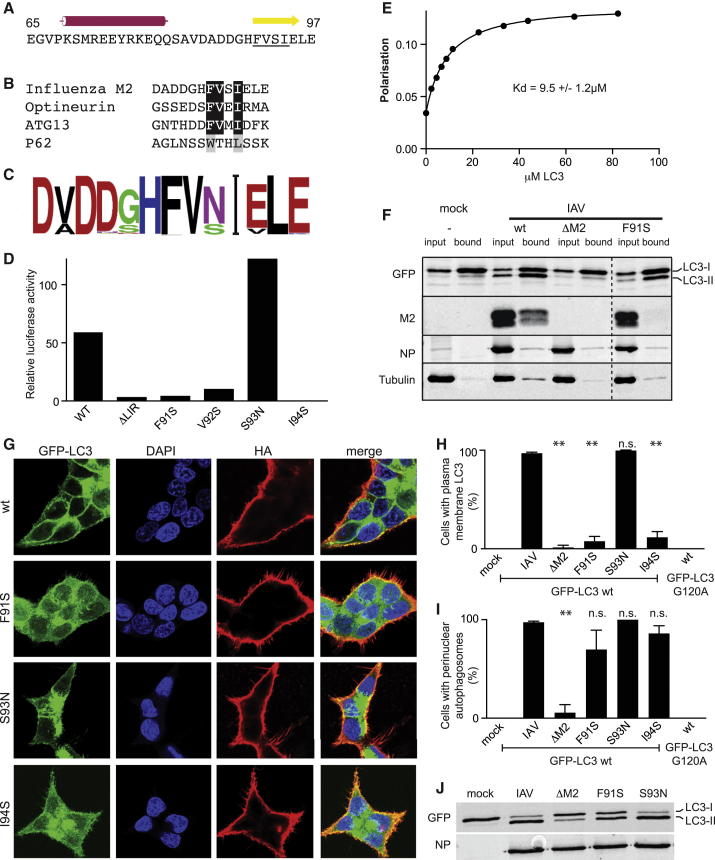
A LIR Motif in Influenza M2 Is Required for LC3 Membrane Localization (A) M2 cytoplasmic tail (from amino acid 65 to C terminus) with predicted α helix and β strand. FVSI motif is underlined. (B) Comparison of M2 LIR motif with established LIRs. (C) Logo derived from 2,685 unique IAV M2 sequences. (D) LUMIER binding assay. Binding of luciferase-tagged cytoplasmic regions of the indicated IAV M2 variants expressed in 293ET cells to beads coated with purified GST or GST-LC3. (E) Fluorescence anisotropy of hydroxycoumarin-labeled M2 C terminus (DADDGHFVSIELE) against purified LC3. (F) Lysates of HCT116 cells expressing GFP-LC3 and infected with the indicated IAVs were immunoprecipitated with GFP-TRAP resin. Input and bound fractions were blotted with the indicated antibodies. Dotted line, irrelevant lanes removed. (G) HCT116 cells expressing GFP-LC3 infected with the indicated virus, fixed at 16 hr p.i., and stained with anti-HA. (H and I) Plasma membrane localization (H) or perinuclear accumulation (I) of GFP-LC3 in HCT116 cells expressing the indicated LC3 alleles and infected with the indicated viruses. Coverslips were assessed blindly in triplicate at 16 hr p.i. Mean and SD. ^∗∗^p < 0.01 by ANOVA. (J) Lysates of HCT116 cells expressing GFP-LC3 were blotted for indicated antigens at 16 hr p.i. with indicated IAV. See also Figures S2 and S3.

Because IAV M2 is required for redistribution of LC3 in infected cells, we speculated that it binds LC3 directly. LIR motifs are β strands that form an intermolecular β sheet on binding to LC3 (Johansen and Lamark, 2011). To identify potential LIRs in M2, we used JPRED to predict secondary structures in its cytosolic tail. One α-helix and one β strand were predicted (Figure 3A). The predicted β strand contains a FVSI motif that matches the consensus LIR motif (Johansen and Lamark, 2011). Among biochemically proven LIR motifs, the M2 FVSI motif most closely corresponds to those found in optineurin and ATG13, which both contain FVxI as their hydrophobic core (Alemu et al., 2012; Wild et al., 2011) (Figure 3B). Preceding the putative M2 LIR motif are three acidic residues, another typical feature of LC3/ATG8 binding proteins.

Strong conservation of motifs frequently indicates biological importance. We therefore examined conservation of the putative M2 LIR motif (Squires et al., 2012). Of 2,685 unique M2 sequences, 2,661 have an FVSI or FVNI motif (Figure 3C). This near-absolute conservation of the hydrophobic residues renders the predicted β strand a strong candidate LIR motif.

To test if IAV M2 binds LC3 via its putative LIR motif, we performed LUMIER binding assays (Barrios-Rodiles et al., 2005; von Muhlinen et al., 2013) with purified GST-LC3 and the cytosolic domain of M2 fused to luciferase. WT M2 and the naturally occurring variant S93N bound LC3, whereas M2ΔLIR, M2 F91S, M2 V92S, and M2 I94S failed to bind (Figure 3D). M2 and the LIR-containing p62 compete for binding to LC3, indicating that M2 binds the same site as the classical p62 LIR (Figure S2A). To determine the monomeric binding affinity of the interaction, a fluorescently labeled peptide corresponding to the last 13 amino acids of M2 and purified LC3 protein were used in fluorescence anisotropy equilibrium measurements. A dissociation constant (K_D_) of 9.5 ± 1.2 μM was determined (Figure 3E). In vivo this interaction would be enhanced by the tetrameric nature of M2, and reduced dimensionality imposed by the presence of both proteins in membranes. To test whether M2 bound LC3 in vivo in a LIR-dependent manner, we performed GFP-trap pull-down experiments in GFP-LC3-expressing cells infected with WT, ΔM2, or M2 F91S IAV. WT M2, but not the M2 LIR mutant F91S, coprecipitated with LC3 (Figure 3F). We conclude that the M2 cytosolic tail contains a conserved LIR motif capable of directly interacting with LC3 in infected cells.

### Plasma Membrane Targeting of LC3 Depends on the M2 LIR Motif

To test whether relocalization of LC3 in infected cells depends on the M2 LIR motif, cells expressing GFP-LC3 were infected with IAV encoding WT or mutant M2 polypeptides (Figures 3G and S2B) and scored for the accumulation of LC3 at the plasma membrane and in perinuclear autophagosomes (Figures 3H and 3I). Both phenomena depended on M2. However, plasma membrane relocalization of LC3 occurred efficiently only in cells infected with viruses encoding functional M2 LIR motifs (WT or M2 S93N), but not in those infected with viruses encoding LIR motifs unable to bind LC3 (M2 F91S and I94S) (Figure 3H). This phenomenon was not due to a delay in replication of mutant viruses (Figure S2C). In contrast, the proportion of cells accumulating perinuclear autophagosomes was not significantly different between WT and LIR mutants (Figure 3I). LC3 unable to become lipidated (LC3 G120A) did not redistribute to either the perinuclear region or plasma membrane in IAV-infected cells (Figures 3H and 3I). Thus, while IAV M2 causes both the accumulation of autophagosomes and the relocalization of LC3 to the plasma membrane, these functions are separable, since the M2 LIR motif is required for the relocalization of LC3 to the plasma membrane, but not its perinuclear accumulation.

Further evidence for M2-mediated but LIR-independent manipulation of the autophagy machinery came from the analysis of LC3 lipidation in infected cells (Figure 3J; also evident in Figure 3F). LC3 undergoes ATG5/ATG12/ATG16-dependent lipidation with phosphatidyl ethanolamine, which provides a membrane anchor to LC3 and is a critical step in autophagosome biogenesis (Ichimura et al., 2000; Kabeya et al., 2000). Lipidated LC3 (LC3-II) migrates faster in SDS-PAGE. Cells infected with WT IAV or the control M2 mutant S93N contained more LC3-II than mock-infected cells, consistent with the virus triggering autophagy (Figure 3J). Enhanced LC3-II levels were dependent on M2 but only partially dependent on the functionality of its LIR motif, as infection with M2 F91S produced intermediate amounts of LC3-II.

To understand how M2 subverts autophagy, cells expressing GFP-LC3 were depleted of FIP200, a component of the ULK kinase complex, or ATG16L1, a core autophagy protein required for LC3 conjugation (Mizushima and Komatsu, 2011) (Figure S3). Depletion of either protein decreased the number of starvation-induced autophagosomes. However, upon infection with IAV, only ATG16L1 was required for the accumulation of perinuclear autophagosomes and the translocation of LC3 to the plasma membrane. These data suggest that influenza stimulates autophagy downstream of FIP200, in a manner distinct from starvation.

Taken together, our data demonstrate that IAV M2 manipulates the autophagy machinery in two separate ways: by causing the accumulation of perinuclear autophagosomes, as previously shown (Gannagé et al., 2009), and by inducing the translocation of LC3 to the plasma membrane. Both phenomena occur in an ATG16L1-dependent and FIP200-independent manner. In contrast, the relocalization of LC3 to the plasma membrane, but not its perinuclear accumulation, requires the LIR-dependent interaction of LC3 with M2.

### Formation of Filamentous Virions Depends on the M2 LIR

**Figure 4:**
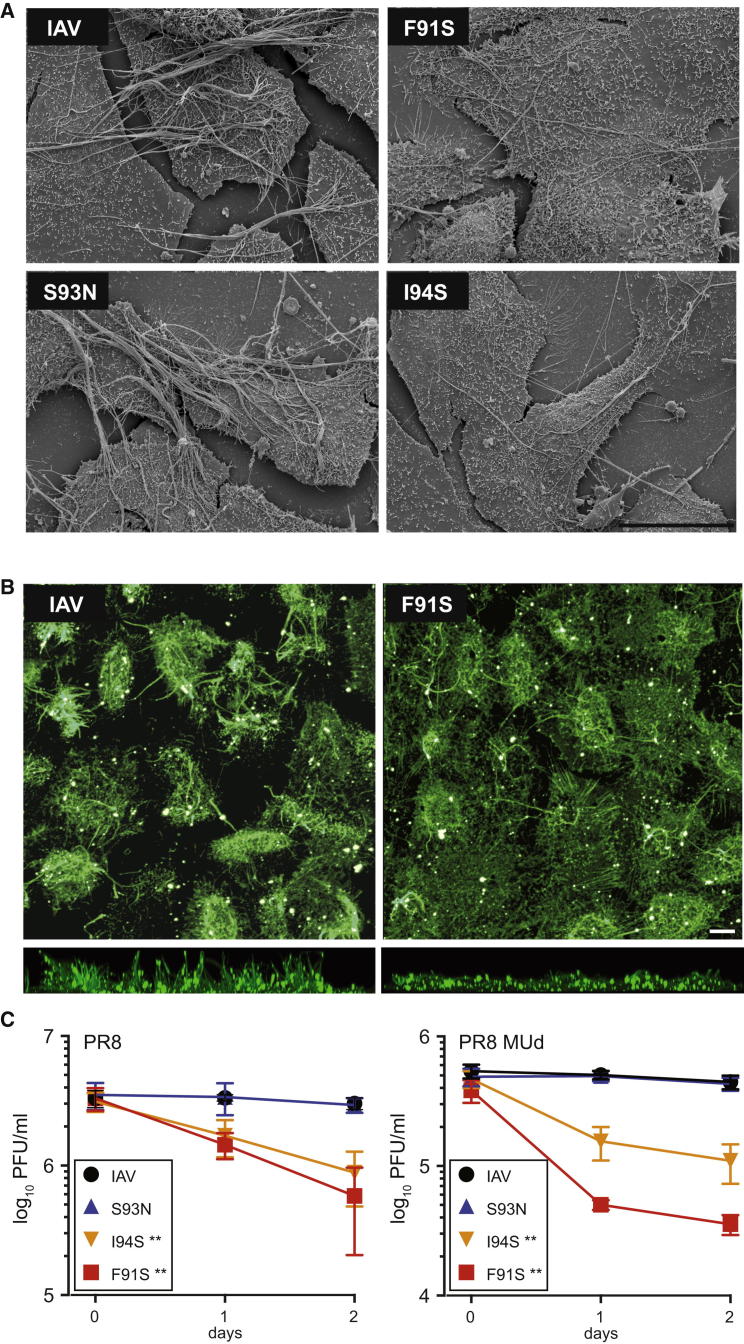
M2 LIR Motif Required for Filamentous Budding and Virus Stability (A) MDCK cells were infected with the indicated PR8 MUd viruses at an moi of 5, fixed at 16 hr p.i., and visualized by scanning electron microscopy. Scale bar, 10 μm. (B) MDCK cells were infected with the indicated PR8 MUd viruses, fixed at 10 hr p.i., and stained without permeabilization with antiserum against PR8 virus. (Top) Maximum intensity z stack projection of confocal images. (Bottom) Side-on view of z stacks. Scale bar, 10 μm. (C) A549 cells infected with WT or mutant PR8 or PR8 MUd viruses at an moi of 1. Cell-free supernatants (16 hr p.i.) were harvested and frozen either immediately or after having been left at room temperature for 1 or 2 days. Experiments were performed in parallel. Titers were determined in triplicate after thawing by plaque assay; mean and standard deviation are shown. ^∗∗^p < 0.01 by ANOVA. See also Figure S4.

The strong conservation of the M2 LIR motif and its requirement for the accumulation of LC3 at the plasma membrane suggest that LC3 relocalization benefits the viral life cycle. IAV can bud in both a spherical and a filamentous fashion, depending on the genetic background of the virus and the host-cell type (Bourmakina and García-Sastre, 2003; Elleman and Barclay, 2004; Roberts and Compans, 1998). Most human-pathogenic isolates of IAV form up to 10 μm long filamentous particles that require extensive membrane resources. Since M2 influences filament formation (Iwatsuki-Horimoto et al., 2006; Roberts and Compans, 1998; Rossman et al., 2010), we hypothesized that accumulation of LC3 at the plasma membrane might represent the virus mobilizing lipid resources for budding. Accordingly, we introduced the M2 LIR mutations into a filamentous strain of IAV, PR8-MUd (Noton et al., 2007). PR8-MUd produces a profusion of micrometer-length filaments arrayed in parallel bundles. While the M2 S93N LIR variant resembled WT virus, cells infected with the M2 F91S or I94S virus encoding nonfunctional LIRs produced fewer filaments with a lower tendency to form bundles (Figure 4A). Confocal microscopy, which biases toward detection of long filament bundles (Simpson-Holley et al., 2002), revealed a large difference in the propensity of cells infected with WT or F91S PR8 MUd to project filament bundles away from the cell surface (Figure 4B). Consistent with a role for autophagy in filamentous budding, both depletion of ATG16L1 and LIR motif mutation reduced the formation of filament bundles (Figure S4). We conclude that the M2 LIR motif is required for normal filamentous budding of IAV.

### Stability of Influenza A Virions Depends on the M2 LIR Motif

To test whether the M2 LIR influences virus replication, we examined growth of the PR8 and PR8 MUd viruses. As expected (Simpson-Holley et al., 2002), the nonfilamentous PR8 virus replicated to higher titers than the filamentous PR8 MUd strain (Figure 4C). Within the two backgrounds, viruses encoding functional and nonfunctional M2 LIR motifs replicated indistinguishably, suggesting the M2 LIR does not directly affect the production of infectious particles (Figures 4C and S2C). However, when viral supernatants were left at room temperature for 24 or 48 hr, viruses encoding WT M2 or M2 S93N remained stable, while viruses encoding nonfunctional LIRs (M2 I94S or F91S) lost infectivity. Filamentous virus was especially sensitive to LIR mutations; PR8 MUd encoding nonbinding M2 LIR mutants suffered a 10-fold drop in titer after just 24 hr at room temperature.

We therefore conclude that the LIR motif in M2 is of functional importance to IAV, as indicated by its high degree of conservation and its essential role in producing stable virions, a prerequisite for the transmissibility of infection.

## Discussion

We describe a functional, highly conserved LC3-binding/LIR motif in the C terminus of IAV M2. This LIR is required for the generation of stable virions, which correlates with the M2 LIR-dependent recruitment of the autophagosomal marker LC3 to the plasma membrane of infected cells. In contrast, the M2 LIR is not required for the induction of autophagy per se, since viruses bearing a mutant M2 LIR enhanced the proportion of lipidated LC3 in infected cells similar to WT IAV. In contrast, viruses lacking M2 scarcely stimulated LC3-II accumulation. We therefore propose a model whereby M2 subverts autophagy by two different means: (1) via a LIR-independent mechanism that, in accordance with a previous report (Gannagé et al., 2009), is not dependent on the M2 cytosolic domain containing the LIR motif, and (2) via the LIR motif identified here, which redirects LC3 to the plasma membrane. The two activities may not be independent, since M2 may stimulate autophagy via beclin-1 (Gannagé et al., 2010) and utilize its LIR motif to transport lipids to the plasma membrane. This mobilization of resources via the autophagy machinery may be important to prevent the depletion of the plasma membrane due to budding virus particles.

The appearance of the autophagosomal marker LC3 at the plasma membrane due to the M2 LIR is an unusual phenomenon, as lipidated LC3 is commonly found only on autophagosomes and their precursors and derivatives, and on phagosomes during LC3-assisted phagocytosis. Although the plasma membrane can contribute to autophagosome biogenesis (Ravikumar et al., 2010), this occurs early in the process and is therefore unlikely to correspond to the IAV-mediated recruitment of LC3 to the plasma membrane reported here. Two distinct mechanisms might target LC3 to the plasma membrane: LC3 may become directly conjugated to the plasma membrane, a scenario that can be demonstrated experimentally but for which no physiological equivalent is known (Fujita et al., 2008), or it may occur by the union of LC3-positive vesicles with the plasma membrane. The only other known physiological instance of LC3 localizing to the plasma membrane occurs in osteoclasts during lysosomal secretion (DeSelm et al., 2011). Our electron micrographs revealed autophagosomes close to the plasma membrane, consistent with the idea that the LC3 at the plasma membrane results from fusion of vesicles. If true, this mechanism suggests that the primary biological purpose of the M2 LIR motif is to deliver appropriate membrane resources for the generation of virus particles. On this account, the M2 protein would ensure both the availability of new transport vesicles by manipulating autophagy downstream of FIP200, and the correct delivery of that lipid resource to the plasma membrane via the LIR motif.

M2 is largely excluded from mature virus particles (Rossman and Lamb, 2011), and immunogold staining for LC3 was observed only rarely in virions. This is consistent with the LIR motif predominantly restoring the depleted plasma membrane of host cells as virus particles bud, rather than contributing directly to the viral membrane. Cells produce up to 10^4^ IAV particles (Möhler et al., 2005). Even if only 1,000 spherical virus particles were produced, their surface corresponds to a substantial fraction (∼20%) of the apical plasma membrane. The far greater amount of membrane required to produce a filamentous virion suggests that, as seen here, LIR function is of particular importance in supporting filament formation. Given that many other RNA viruses (especially respiratory pathogens) produce filamentous particles (Bächi and Howe, 1973; von Magnus, 1953; Yao and Compans, 2000), it may be that other enveloped viruses have also evolved mechanisms to mobilize lipid resources from host cells via autophagy.

Transmission of infection between individuals and species depends on survival of virions outside the host. The relative contribution of respiratory droplets, fomite, and aerosol transmission for IAV is still under debate, but the fomite route in particular is a time-demanding process (Brankston et al., 2007; Hall, 2013). Significantly, IAV with mutations in its LIR is less stable. We have demonstrated that filament formation is affected by LIR mutations. Ultrastructural studies of viruses lacking the M2 C terminus (and thus the LIR motif) showed that even spherical virions had decreased structural integrity (Sugita et al., 2011). We conclude that the LIR motif is involved in budding and is required for morphologically normal and stable viral progeny.

Whereas decreased stability is detrimental to the spread of the virus, it may have a technological use. There are significant concerns surrounding the manipulation of highly pathogenic strains of IAV, especially in potential gain-of-function experiments (Langlois et al., 2013; Patterson et al., 2013). LIR mutations could potentially provide enhanced biosafety by ensuring decreased environmental stability.

This study reveals that IAV utilizes a SliM to hijack autophagy. How this process enhances virion stability remains open, but it likely involves delivery of appropriate resources to the plasma membrane during budding, as evidenced by the highly unusual appearance of LC3 at the plasma membrane. The economy with which the LIR motif plays its role suggests that other enveloped viruses may have evolved similar motifs, and it is possible that the discovery of LIRs in other viruses will provide new targets for intervention.

## Experimental Procedures

### Viruses

Recombinant IAVs were rescued by eight plasmid transfection and segment seven, verified by sequencing (Wise et al., 2012). Cells were infected by allowing virus to adsorb for 30–60 min in serum-free medium. Plaque assays were carried out in MDCK cells overlaid with Avicel and stained with toluidine blue. Growth kinetics were determined by performing a plaque assay on the initial inoculum, which was then removed by acid washing. The medium was replaced by SFP supplemented with 0.14% BSA and 1 μg/ml TPCK trypsin, and further plaque assays were performed at different time points postinfection.

### Cells

Cells were maintained in IMDM or DMEM supplemented with 10% FCS and gentamicin. HCT116 and A549 cells were transduced with MLV-based retroviruses encoding GFP-LC3 (Randow and Sale, 2006).

### Antibodies

Antibodies were from Abcam (M2-14/C2, NP-AA5H, GFP-JL/8), and the antiserum against NP was described by Noton et al. (2007).

### Protein Assays

Immunoprecipitation, western blot, and LUMIER assays were performed as described previously (Barrios-Rodiles et al., 2005; von Muhlinen et al., 2012; Ryzhakov and Randow, 2007). For fluorescence anisotropy, LC3 was expressed in *E. coli* and purified as described (von Muhlinen et al., 2012). GFP-trap experiments (Chromotek) were performed at 16 hr postinfection according to the manufacturer’s protocols. Fluorescence measurements of LC3, serially diluted and mixed with 100 nM hydroxycoumarin-labeled M2 LIR peptide, were performed on a Cary Eclipse fluorescence spectrophotometer (Agilent) in triplicate.

### Microscopy

For fluorescence microscopy, cells were grown on glass coverslips. Confocal images were taken with a 63×, 1.4 numerical aperture objective on a Zeiss 780 microscope. For TEM immunogold labeling, appropriately transduced cells were fixed at 16 hr p.i. with IAV, permeabilized, and sequentially labeled with rabbit anti-GFP and goat anti-rabbit ultrasmall gold (Aurion 100.011), followed by silver enhancement and fixation with osmium tetroxide. Ultrathin sections were examined on a Philips 208 EM at 80 kV. For SEM visualization of viral filaments, cells were coated with 10 nm of gold and viewed on an FEL-Philips XL30 FEGSEM at 5 kv.

### siRNA

A549 cells expressing GFP-LC3 were transfected on days 1 and 3 with siRNA and were challenged with either infection or starvation on day 5.

## Supplemental Information

Document S1. Figures S1–S4 and Supplemental Experimental Procedures
