# Supplementary material for: A LC3-Interacting Motif in the Influenza A Virus M2 Protein Is Required to Subvert Autophagy and Maintain Virion Stability
Source: Cell Host Microbe. 2014 Feb 12;15(2):239–47. doi: 10.1016/j.chom.2014.01.006 (PMC3991421; doi:10.1016/j.chom.2014.01.006)
Supplement: Document S1. Figures S1–S4 and Supplemental Experimental Procedures [file mmc1.pdf]

Cell Host & Microbe, Volume *15*

Supplemental Information

A LC3-Interacting Motif in the Influenza A Virus M2 Protein Is Required to Subvert Autophagy and Maintain Virion Stability

Rupert Beale, Helen Wise, Amanda Stuart, Benjamin J. Ravenhill, Paul Digard, and Felix Randow

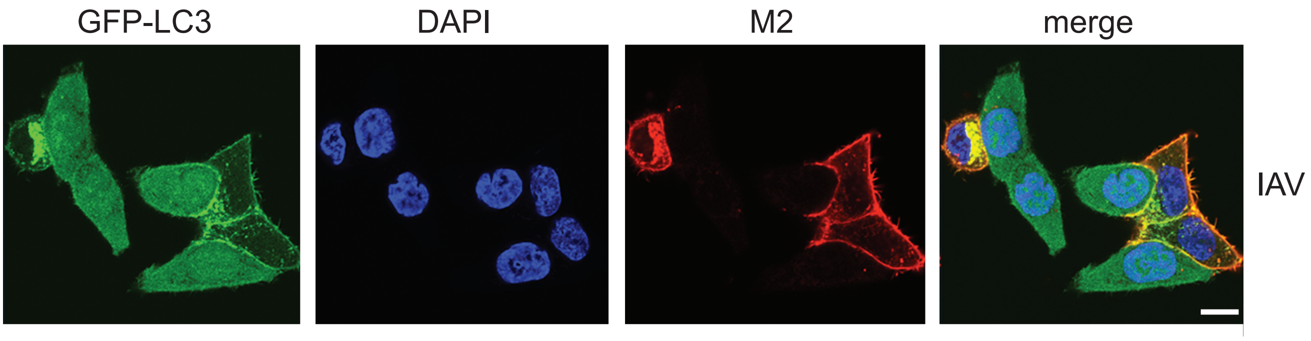

Supplemental Figure 1

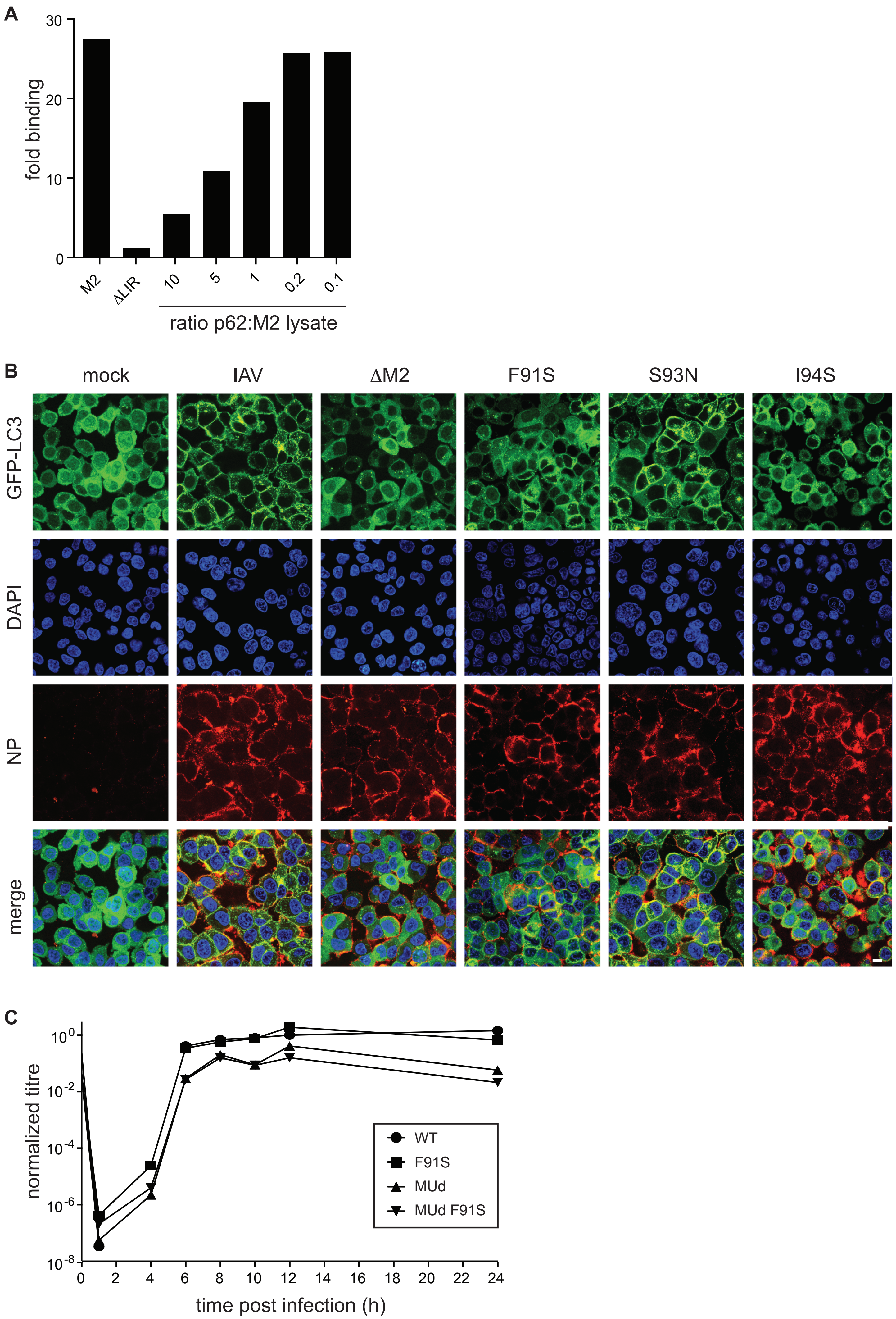

Supplemental Figure 2

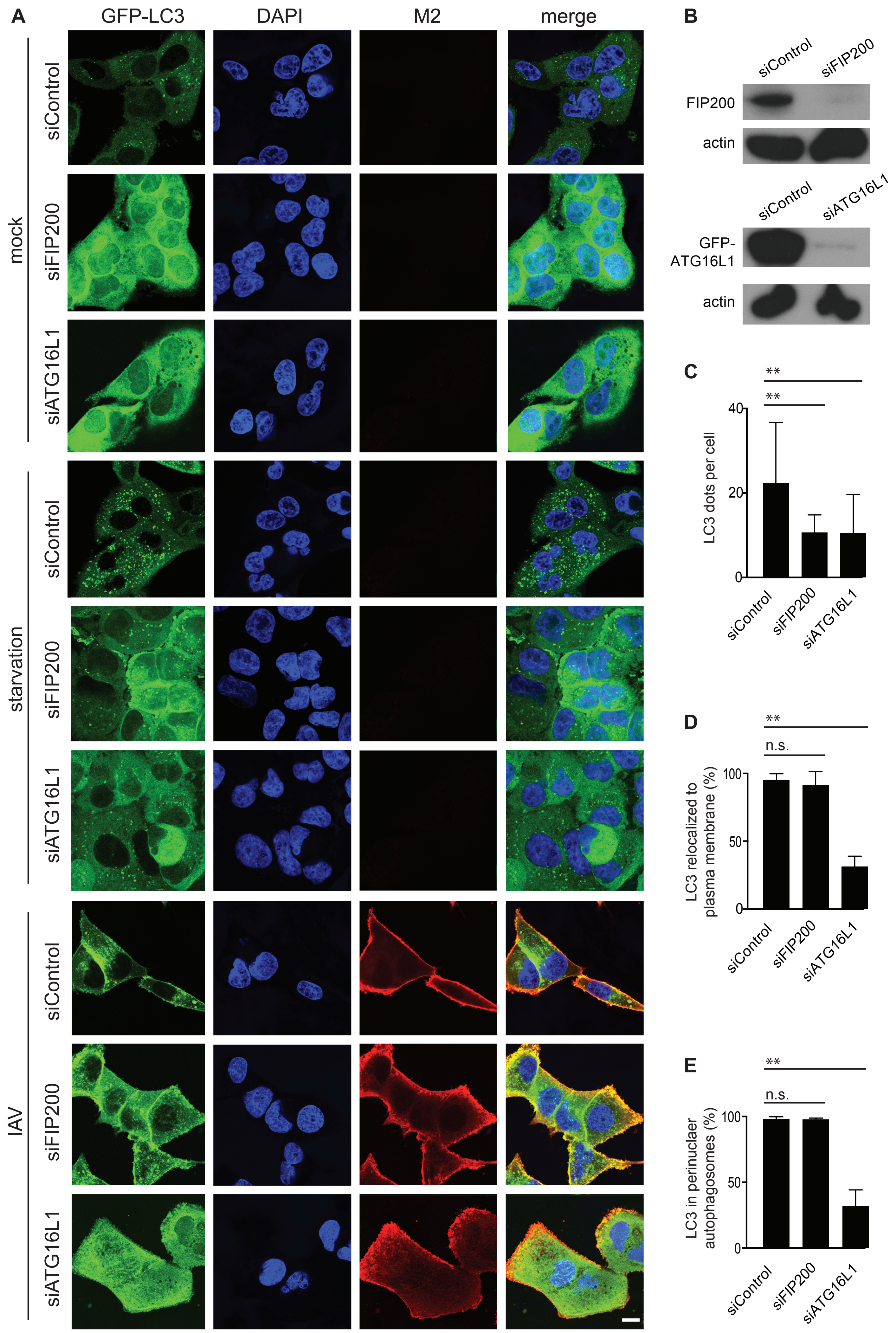

Supplemental Figure 3

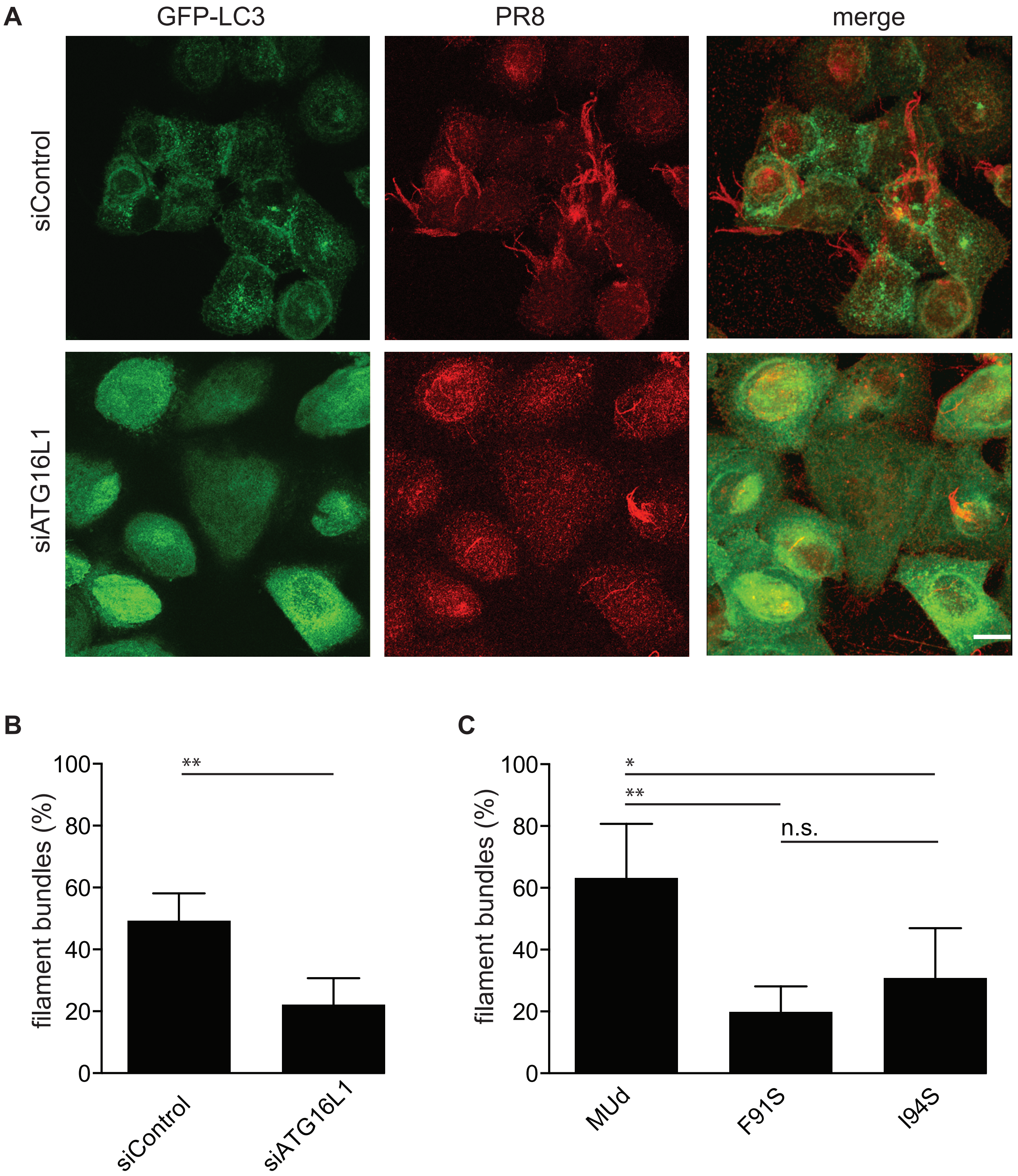

Supplemental Figure 4

Supplemental Figures Legends

Figure S1, related to Figure 1

Human epithelial HCT116 cells expressing GFP-LC3 were infected with IAV at an MOI of 0.2, fixed at 16 hours p.i. and stained for M2. Scale bar 10µm.

Figure S2, related to Figure 3

A) LUMIER binding assay. Binding of luciferase-tagged cytoplasmic region of M2 expressed in 293ET cells to beads coated with purified GST-LC3 relative to GST. Lysate of 293ET cells expressing mCherry-p62 was added as indicated.

B) Human lung epithelial A549 cells expressing GFP-LC3 were infected with IAV or the indicated mutants at an MOI of 5, fixed at 16 hours p.i. and stained for NP. Scale bar 10µm.

C) Single-cycle growth kinetics of PR8 and MUd. Titres were determined by plaque assay at the indicated times after infection. Relative PFU/ml calculated in comparison to the experimentally determined titre of the input.

Figure S3, related to Figure 3

A) A549 cells expressing GFP-LC3 were transfected with either the indicated siRNAs and either mock-infected, starved or infected with IAV at an MOI of 5. Cells were fixed and stained for M2 at 16 hours p.i. or after one hour of starvation. Scale bar 10µM.

B) Wild type or GFP-ATG16L1 expressing A549 cells, treated with the indicated siRNAs, lysed and probed with the indicated antibodies.

C) The number of GFP-LC3 punctae in A549 cells subjected to the indicated siRNA treatment and starved for 1 hour. Mean and standard deviation of triplicates (30 cells per coverslip), **p=< 0.01 by ANOVA.

D) Fraction of A549 cells with GFP-LC3 plasma membrane localization 16 hours p.i. with IAV at an MOI of 5. Mean and standard deviation of triplicates (100 cells per coverslip), **p<0.01 by ANOVA

E) Fraction of A549 cells with perinuclear GFP-LC3 accumulation 16 hours p.i. with IAV at an MOI of 5. Mean and standard deviation of triplicates (100 cells per coverslip), **p<0.01 by ANOVA

Figure S4, related to Figure 4

A) Confocal Z-stack images of A549 cells expressing GFP-LC3, treated with the indicated siRNAs and infected with filamentous (MUd) virus at an MOI of 5, fixed at 16 hours p.i. and stained with anti-PR8 antiserum. Leftmost two panels show the confocal images with the greatest number of filament bundles, right panel shows the maximum intensity projection of the whole Z-stack. Scale bar 10μm.

B) Fraction of GFP-LC3 expressing A549 cells with at least one filament bundle of greater than 5μm at 16h p.i. with MUd virus. Mean and standard deviation of triplicates (100 cells per coverslip), **p<0.01 by t-test.

C) Fraction of GFP-LC3 expressing A549 cells with at least one filament bundle longer than 5μm at 16h p.i. with the indicated MUd viruses. Mean and standard deviation of triplicates (100 cells per coverslip), **p<0.01, *p<0.05 by ANOVA.

Supplemental experimental procedures

Viruses, Cells, Plasmids and Antibodies.

Recombinant viruses were rescued by 8 plasmid transfection into 293T cells followed by amplification in Madin-Darby Canine Kidney (MDCK) cells as previously described (Hutchinson et al., 2008). PR8 was rescued using the system described and generously provided by (de Wit et al., 2004). PR8-MUd has been previously described (Noton et al., 2007). A549 human lung adenocarcinoma and HCT116 human colon adenocarcinoma cells were transduced with MLV-based retroviruses encoding GFP-LC3 (Randow and Sale, 2006). Cells were cultured according to standard procedures, and were infected by allowing virus to adsorb for 30–60 min in serum free medium. Plaque assays were carried out in MDCK cells using an Avicel overlay followed by staining with toluidine blue (Hutchinson et al., 2008). Growth kinetics were determined by performing a plaque-assay on the initial inoculum, which was then removed by acid washing. The medium was replaced by SFP supplemented with 0.14%BSA and 1μg/ml TPCK trypsin, and further plaque assays were performed at different time-points post infection. To detect IAV M2, mouse monoclonal antibody clone 14/C2 (Abcam) was used. To detect IAV NP, either monoclonal AA5H (Abcam) or a rabbit polyclonal serum raised against whole NP (A2915) (Noton et al., 2007) were used. To stain the surface of virus infected cells, a rabbit polyclonal serum raised against whole PR8 virions was used (Amorim et al., 2007). A mouse monoclonal to GFP (clone JL/8) was sourced from Abcam.

Structural prediction and sequence logo

The JPRED algorithm was used to identify putative structural features in the

C-terminus of M2 (Cole et al., 2008). The sequence logo was compiled by identifying all unique M2 sequences in the database and the C-terminal 13 amino-acids were fed into the WebLogo 3 program (Schneider and Stephens, 1990).

Binding assays

Immunoprecipitation, Western Blot, and LUMIER assays were performed as described previously (Barrios-Rodiles et al., 2005; Ryzhakov and Randow, 2007). For fluorescence anisotropy, LC3 was expressed in *E. coli* and purified as described (Muhlinen et al., 2012). GFP-trap experiments (Chromotek) were performed at 16 hours post infection according to the manufacturer's protocols (Amorim et al., 2011). Fluorescence measurements of LC3, serially diluted and mixed with 100 nM hydroxycoumarin-labeled M2 LIR peptide, were performed on a Cary Eclipse fluorescence spectrophotometer (Agilent) in triplicate. Binding data was analyzed with GraphPad Prism 6.

Microscopy

For fluorescence microscopy, cells were grown on glass coverslips and fixed in 4% paraformaldehyde or formaldehyde. They were incubated with primary, followed by secondary antibodies and/or Wheat-Germ agglutinin (Invitrogen) before being mounted. Confocal images were taken with a x63, 1.4 numerical aperture objective on a Zeiss 780 microscope. For TEM immunogold labeling, appropriately transduced HCT116 cells were grown on glass bottom petri dishes (MatTek) and infected at an MOI of 5. 16 hours after infection, cells were fixed overnight at 4°C. Cells were permeabilised and incubated with rabbit anti-GFP antiserum then incubated with goat anti rabbit ultrasmall gold (Aurion 100.011) overnight at 4°C. After silver enhancement and fixation with

osmium tetroxide, cells were dehydrated and embedded in resin. Ultrathin sections were examined using a Philips 208 EM operated at 80kV. For SEM visualization of viral filaments, cells were fixed, rinsed and treated with 2% uranyl acetate. They were dehydrated, then glued to SEM stubs with colloidal silver. The stubs were coated with 10nm of gold and viewed in an FEL-Philips XL30 FEGSEM at 5kv.

siRNA

A549 cells expressing GFP-LC3 were transfected with siRNAs specific to ATG16L1 (GGCUCUGCUGAGGGCUCUCUGUAUA) (Muhlinen et al., 2012), FIP200 (CAGUAAUGGCCAAGAUUCCACUGUU) or a non-targeting control (Invitrogen) using Lipofectamine 2000 on both day 1 and day 3. Cells were then either infected or starved by transfer to EBSS media for one hour on day 5 (when optimal inhibition of starvation-induced autophagy was observed).

Supplemental References

Amorim, M.J., Bruce, E.A., Read, E.K.C., Foeglein, A., Mahen, R., Stuart, A.D., and Digard, P. (2011). A Rab11- and microtubule-dependent mechanism for cytoplasmic transport of influenza A virus viral RNA. Journal of Virology *85*, 4143–4156.

Amorim, M.J., Read, E.K., Dalton, R.M., Medcalf, L., and Digard, P. (2007). Nuclear export of influenza A virus mRNAs requires ongoing RNA polymerase II activity. Traffic *8*, 1–11.

Barrios-Rodiles, M., Brown, K.R., Ozdamar, B., Bose, R., Liu, Z., Donovan, R.S., Shinjo, F., Liu, Y., Dembowy, J., Taylor, I.W., et al. (2005). High-throughput mapping of a dynamic signaling network in mammalian cells. Science *307*, 1621–1625.

Cole, C., Barber, J.D., and Barton, G.J. (2008). The Jpred 3 secondary structure prediction server. Nucleic Acids Research *36*, W197–W201.

de Wit, E., Spronken, M.I.J., Bestebroer, T.M., Rimmelzwaan, G.F., Osterhaus, A.D.M.E., and Fouchier, R.A.M. (2004). Efficient generation and growth of influenza virus A/PR/8/34 from eight cDNA fragments. Virus Res *103*, 155–161.

Hutchinson, E.C., Curran, M.D., Read, E.K., Gog, J.R., and Digard, P. (2008). Mutational analysis of cis-acting RNA signals in segment 7 of influenza A virus. Journal of Virology *82*, 11869–11879.

Muhlinen, von, N., Akutsu, M., Ravenhill, B.J., Foeglein, A., Bloor, S., Rutherford, T.J., Freund, S.M.V., Komander, D., and Randow, F. (2012). LC3C, Bound Selectively by a Noncanonical LIR Motif in NDP52, Is Required for Antibacterial Autophagy. Molecular Cell *48*, 329–342.

Noton, S.L., Medcalf, E., Fisher, D., Mullin, A.E., Elton, D., and Digard, P. (2007). Identification of the domains of the influenza A virus M1 matrix protein required for NP binding, oligomerization and incorporation into virions. J. Gen. Virol. *88*, 2280–2290.

Randow, F., and Sale, J.E. (2006). Retroviral transduction of DT40. Subcell Biochem *40*, 383–386.

Ryzhakov, G., and Randow, F. (2007). SINTBAD, a novel component of innate antiviral immunity, shares a TBK1-binding domain with NAP1 and TANK. Embo J *26*, 3180–3190.

Schneider, T.D., and Stephens, R.M. (1990). Sequence logos: a new way to display consensus sequences. Nucleic Acids Research *18*, 6097–6100.
